# Dispersing upconversion nanocrystals in a single silicon microtube

**DOI:** 10.1038/srep35941

**Published:** 2016-10-25

**Authors:** Hanyang Li, Yan Wang, Hui Li, Yundong Zhang, Jun Yang

**Affiliations:** 1 Key Lab of In-fiber Integrated Optics, Ministry Education of China, Harbin Engineering University, Harbin 150080, China; 2 National Key Laboratory of Tunable Laser Technology, Institute of Opto-Electronics, Harbin Institute of Technology, Harbin 150080, China

## Abstract

Nanocrystals of Ln^3+^ (Ln = Yb, Tm and Ho) doped *β*-NaLuF_4_ with average diameter about 200 nm are dispersed in silica-based microtube (MT) by a simple flame heating method. The fabricated microtube has a diameter range from 2 μm to 30 μm and lengths up to hundreds microns. The fluorescence of upconversion nanocrystals (UCNCs) can propagate along a single MT and couple into another MT through evanescent field. The guiding performance of the single UCNCs doped MT is measured to prove that it can be used as an active waveguide. Moreover, optical temperature sensing based on the single UCNCs-MT is also demonstrated, and the sensitivity of UCNCs-MT is significantly enough for thermometry applications in the range of 298–383 K.

Lanthanide doped upconversion nanocrystals (UCNCs) have been investigated extensively due to their tremendous potential applications such fields as multimodal bioimaging^1^,^2^,^3^, electro-optical modulator^4^,^5^, magnetic sensors^6^,^7^, and optical temperature sensing^8^,^9^,^10^. Compared with traditional organic fluorophores or semi-conductor quantum dots (QDs), UCNCs show superior features, such as high photochemical stability, non-blinking, resistance to photobleaching, absence of autofluorescence, and tissue-penetrable near-infrared light excitation^11^,^12^,^13^. At present, most studies have focused on the synthesized Ln^3+^ doped host materials, including nanorods^14^, spherical nanoparticles^15^, tetragonal and hexagonal nanoparticles^16^,^17^. Take the optical thermometry experiment for example; the UCNCs powder is easy oxidized at a high temperature which may limit the application of the UCNCs. One strategy to overcome oxidation is the use of core/shell architectures where a shell of SiO_2_ material grows around the UCNCs^18^. However, the UCNCs are difficult to be directly integrated on the chip as a micro structure optical sensor.

One-dimensional fluorescence micro-wave guide, such as dye-doped polymer nanowire and QDs doped nanofiber, have been studied and applied to a series of integrated microstructure optical devices, including nano lasers, micro-optical sensors, fiber biosensors^19^,^20^,^21^,^22^,^23^. The fluorescence photons can be coupled into guided modes of microfibers or nanofibers due to the high efficiency evanescence^24^. However, limited attention has been paid to integration of UCNCs in microstructure optical waveguide, such as silica based microfiber. The UCNCs have unique potential advantages, such as near infrared (NIR) light excitation and visible light emissions. Therefore, integrating UCNCs into micro or nanoscale wave guide is possible to take for the biophotonic application. Owing to the strong tissue penetration, NIR photons were absorbed by rare earth ions in a high efficiency^25^.

Here, we report a simple method to doped *β*-NaLuF_4_: Yb^3+^/Tm^3+^ and *β*-NaLuF_4_: Yb^3+^/Ho^3+^ UCNCs on the inner surface of microtube (Tm^3+^/Ho^3+^-UCNCs-MT). Under 980 nm laser exciting, the upconverted emission propagated along the wall of the MT. Moreover, the upconversion dynamic performance of Tm^3+^-UCNCs-MT dependent on two excitation modes was compared. We also demonstrated optical thermometry based on the single MT due to the unique electron configuration of lanthanide active ions.

## Results

### Upconversion nanocrystals characterization

**Figure 1:**
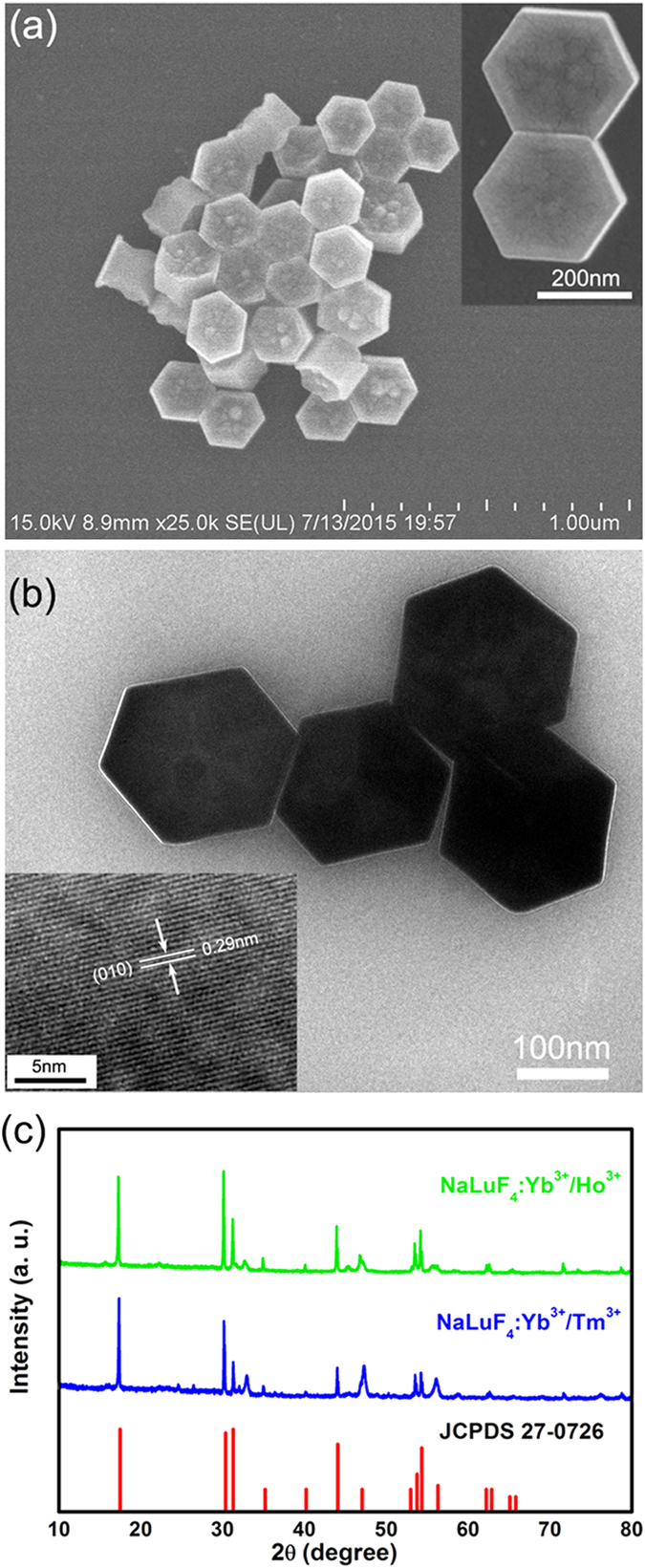
Microscopic characterization of UCNCs. (**a**) SEM image of UCNCs, the inset is a magnified image. (**b**) TEM image of UCNCs, the inset is a HRTEM image. (**c**) XRD measurement of *β*-NaLuF_4_: Yb^3+^/Tm^3+^ and *β*-NaLuF_4_: Yb^3+^/Ho^3+^ nanocrystals.

Figure 1a shows the scanning electron microscope (SEM) image of the UCNCs. The UCNCs exhibit a uniform six-sided prismatic structure with monodispersity and good crystallinity. The prismatic structure has an average diameter of 200 nm and a thickness of 100 nm. Figures 1b is the typical transmission electron microscopy (TEM) image, which shows the obvious hexagonal microstructure with uniform size distribution. The inset is a high-resolution transmission electron microscopy (HRTEM) image, and the lattice fringes in the HRTEM image confirm the crystallinity. The distance of 0.29 nm between the adjacent lattice fringes corresponds to the d_110_ spacing of the hexagonal NaLuF_4_ phase^17^. To investigate the crystal structure and phase purity, the UCNCs were characterized by XRD. All samples obtained in our experiments show similar results, with the XRD patterns of UCNCs presented in Fig. 1c. All the observed diffraction peaks of the nanocrystals can be perfectly indexed to the tetragonal phase of *β*-NaLuF_4_ (JCPDS No. 27-0726) and no other diffraction peaks from any impurity were found.

### UCNCs-MT fabrication and optical characterization

**Figure 2:**
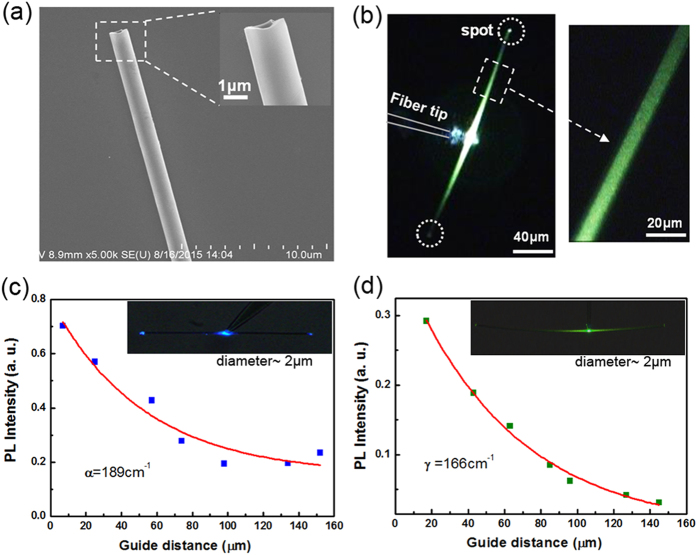
Guiding performance of the UCNCs-doped MT. (**a**) SEM images of UCNCs-doped MT. The inset is close-up view of the MT and the scalebar is 1 μm. (**b**) True-color microscope image of a Ho^3+^-UCNCs-MT with diameter of 7 μm and lengths of 200 μm and part of the MT luminous image. (**c**) Relationship of normalized PL spectra intensity and guiding distance (*d*), the inset is PL microscope image for Tm^3+^-UCNCs-MT with diameter of 2 μm. (**d**) Relationship of PL intensity and *d*, the inset is PL microscope image for Ho^3+^-UCNCs-MT with diameter of 2 μm.

The UCNCs-MT used in our experiment are fabricated by preheat and flame-heated drawing method. The preheating process eliminated the air in the microtube to protect the UCNCs powder from oxidation during the hydrogen flame drawing process and beneficial to get uniform UCNCs-MT (see Supplementary Fig. S1). Figure 2a shows the SEM images of one UCNCs-MT after the drawing process, and we obtained MTs with outside diameters down to 2 μm; wall thickness down to 400 nm and lengths up to hundreds microns. The close-up view of the MT (inset of Fig. 2a) indicates that the MT has a uniform diameter and smooth sidewall. The UCNCs-doped MTs were cut and manipulated by two micromanipulators (i.e. a tapered fiber tip is stabilized on a resolution of 0.1 μm XYZ translation stage) under optical microscope.

To investigate the optical properties, one single UCNCs-MT was deposited on an MgF_2_ substrate (n = 1.39). The 980 nm continuous laser was coupled into a fiber tip which was used to excite the MT through high efficiency evanescent^26^ (see Supplementary Fig. S2). Figure 2b shows the dark field microscope (40× objective) image of a Ho^3+^-UCNCs-MT, (diameter ~7 μm, lengths ~200 μm) where exciting point was located in the middle of MT. Moreover, two bright spots were observed at both ends of the Ho^3+^-UCNCs-MT, which serves as a typical feature of an optical waveguide, suggesting that the MT absorbs the light and propagates PL toward the ends^27^. A part of this Ho^3+^-UCNCs-MT luminous image (60× objective) was shown in Fig. 2b, and the tiny spots of brightness proved that the UCNCs distribution within MT is uniform on this large diameter scale. There is no evidence of appreciable scattering centers such as break points or UCNCs clustering (see Supplementary Information Fig. S3).

The power of the excitation light outgoing from the tapered fiber tip was set as low as 0.56 mW to clearly observe the decay effect of the MT. Figure 2c shows the relationship of normalized photoluminescence (PL) spectra intensity and propagation distance, with the inset being a dark-filed PL microscope image of this Tm^3+^-UCNCs-MT (diameter ~2 μm). Here, by studying the image brightness, as used in previous work^28^,^29^, the normalized PL intensity of the emission light can thus be calculated. The PL intensity of the output spot is normalized against the excited spot, and then the decay of the guided normalized PL intensity dependent propagation distance (*d*) is obtained. According to Lambert-Beer law, the normalized intensity was fit by first order exponential decay as the red line shown in Fig. 2c; with *d* increases, the PL intensity decreases as ≈ exp (−α*d*), and the loss coefficient is α = 189 cm^−1^. The guiding performance of Ho^3+^-UCNCs-MT was also measured (i.e. excitation power is 0.56 mW). The inset of Fig. 2d shows PL microscope image of Ho^3+^-UCNCs-MT with diameter of 2 μm. Besides, Fig. 2d also shows the decay curves and the loss coefficient is γ = 166 cm^−1^. The waveguiding properties of MT with different diameters were also investigated (see Supplementary Figs S4–S7). The loss coefficient α and γ have a little difference also the MT have a same diameters (~2 μm), which may be caused by the difference up-conversion luminescence intensity between Tm^3+^ and Ho^3+^. The mechanisms affecting optical losses in the UCNCs-MT are mainly due to self-absorption and Rayleigh scattering. The absorption cross section of Ln^3+^ ions has a large overlap with the emission cross section lead to the Ln^3+^ ions have serious fluorescence self-absorption^30^. The UCNCs with dimension smaller than the wavelength lead to the Rayleigh scattering. The measured loss coefficient of the UCNCs-MT is agreed with CdSe/ZnS-doped nanofiber (247 cm^−1^)^31^ and is little smaller than that of electrospun micro-fibers (355 cm^−1^)^32^ which are typical characteristic of active wave guides. However, the absorption coefficient seems extremely large compared with the passive waveguide, such as silica nanofiber with ultra-lowe-loss (10^−2^ dB/mm)^33^.

**Figure 3:**
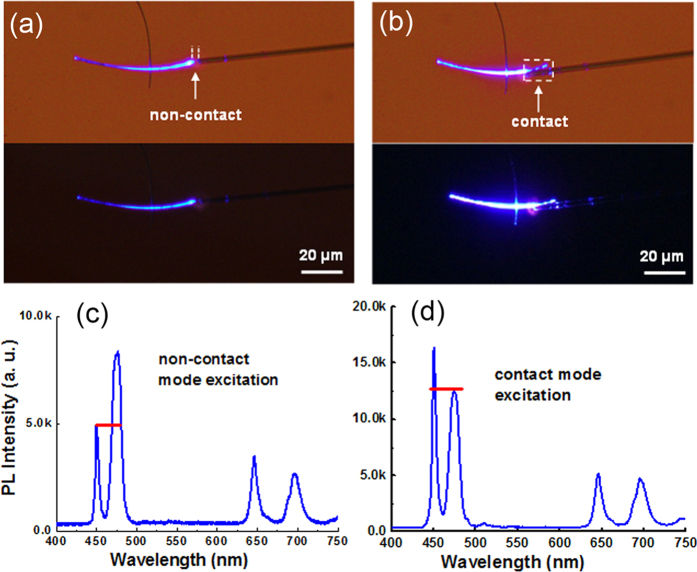
Optical properties of the UCNCs-MT. (**a**) Microscope images for one single Tm^3+^-UCNCs-MT with diameter of 2 μm and lengths of 60 μm was excited by non-contact mode. (**b**) Microscope images for the Tm^3+^-UCNCs-MT was excited by contact mode. (**c**) The PL spectrum of the Tm^3+^-UCNCs-MT under the contact mode excitation and (**d**) non-contact mode excitation.

One single Tm^3+^-UCNCs-MT (diameter ~2 μm, lengths ~60 μm) was excited under the same excitation laser power of 0.6 mW, but in different modes. One is non-contact mode that a short distance (~5 μm) exists between the fiber tip and the MT endpoint, as shown in Fig. 3a. Another is contact mode that the fiber tip is in contact with the excited MT, and the connection distance is about 15 μm, as shown in Fig. 3b. Tm^3+^-UCNCs-MT has a stronger luminescence and higher evanescent coupling efficiency in contact mode excitation. In addition, through the contact mode, the emissions can be coupled into another MT (diameter ~2 μm) which was placed under the excited Tm^3+^-UCNCs-MT, and we found that when the cross angle between two MTs is 90° the coupling efficiency is low as shown in Fig. 3b. However, the evanescent wave coupling efficiency can be improved by tuning the cross angle between two MTs^34^ (see Supplementary Fig. S8).

The PL spectrum of this Tm^3+^-UCNCs-MT (in Fig. 3a,b) was recorded and shown in Fig. 3c,d under the contact mode, the emission intensity at λ = 450 nm (*I*_450_) is 1.3 times larger than the intensity at λ = 476 nm (*I*_476_). Conversely, under the non-contact mode, the *I*_450_/*I*_476_ is only 0.58. As we known, the relationship between n photons processes of upconversion luminescence intensity and power is described as *I*_f_ ∝ *P*_n_, where *I*_f_ is up conversion luminescence intensity, *P* is excitation laser power, and n is the number of photons absorbed per up converted photon emitted^35^. There is 2–3 photons absorbed process for λ = 450 nm emitted and 1–2 photons absorbed process for λ = 476 nm corresponding to the ^1^D_2_ → ^3^F_4_ and ^1^G_4_ → ^3^H_6_ transitions, respectively (see Supplementary Fig. S9). It is noted that the value of n can be different by changing excitation laser intensity. In Yb^3+^-Tm^3+^ co-doped systems, different multi-step excited state absorption processes may result in different n photons absorbed. For example, at low excitation density range, up conversion luminescence could be 2 absorbed photons process, while it could be 3 absorbed photons process at high excitation density range, because the distribution of the population on the intermediate energy level may be different^36^ (see Supplementary Fig. S10).

### Optical-thermal properties of UCNCs-MT

**Figure 4:**
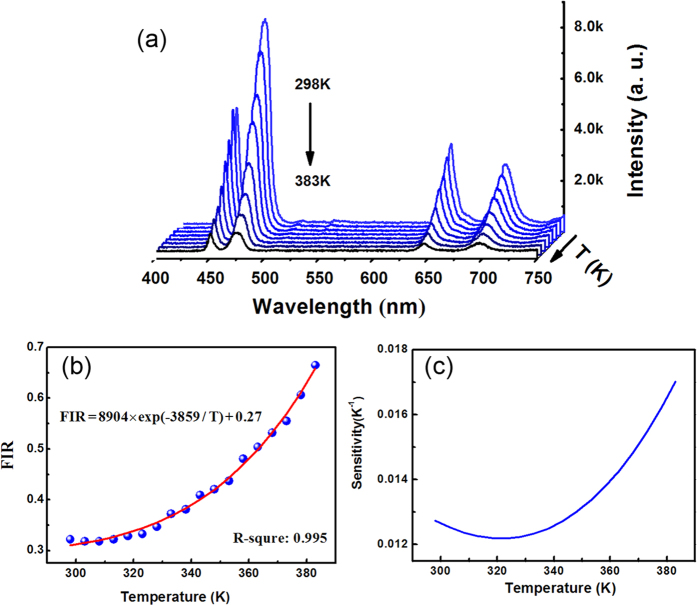
Optical-thermal properties of the Tm^3+^-UCNCs-MT. (**a**) Emission spectra of Tm^3+^-UCNCs-MT in the range from 400 to 750 nm measured at a series of temperatures between 298 and 383 K. (**b**) The relationship between the FIR and the temperature for Tm^3+^-UCNCs-MT. (**c**) The sensing sensitivity as a function of temperature for Tm^3+^-UCNCs-MT.

The optical-thermal properties of Tm^3+^ and Ho^3+^ emissions from the single MT were studied. Figure 4a shows the emission spectra of Tm^3+^-UCNCs-MT in the range from 400 to 750 nm. They were measured at a series of temperatures between 298 and 383 K (acquisition of spectrum every 5 °C) and the power of the excitation light was set as low as 0.56 mW to reduce the heating effect generated by the excitation light. It is observed that the intensities emissions are decreased with the rise of temperature. When temperature was higher than 388 K, the fluorescence intensity was too weak to detect. On the other hand, the coupling point of fiber tip to MT can be slightly changed if the temperature higher than 398 K, caused by the heating of air flow. It is observed that the intensities emissions are decreased with the rise of temperature. For Tm^3+^-UCNCs-MT, the emission intensity of 475 nm would experience a sharp decline while that of 698 nm decreased slowly. To confirm the different decay rates were caused by temperature changes, the Tm^3+^-UCNCs-MT was emitting until the temperature is reduced from 383 to 298 K. It was found that the emission intensity was back to the original location in spectrum. In fact, the UCNCs powder pumped by focusing 980 nm laser could make an effective optical thermometer in a large temperature range such as 100–700 K^18^.

The fluorescent intensity ratio (FIR) technique was used to examine the optical temperature sensing of Tm^3+^-UCNCs-MT in our experiments and this technique has been successfully demonstrated in a variety of rare earth doped systems^37^. Accordingly, the FIR from each thermally coupled level of active ions can be defined by the following equation^38^:


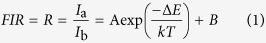


where, *I*_*a*_and *I*_*b*_are intensities of emission from the upper and the lower thermally coupled levels, A and B are constants, in which A depends on the spontaneous emission rate, degeneracy and emission energies; besides, Δ*E* is the energy difference between thermally coupled levels, *k* is the Boltzmann constant and *T* is the temperature.

In the case of Tm^3+^-UCNCs-MT, the emission intensities of two peaks *I*_476_ and *I*_697_ corresponding to the energy level transition are ^1^G_4_ → ^3^H_6_ and ^3^F_2,3_ → ^3^H_6_. The relationship between the FIR and the temperature in the region of 298–383 K is shown in Fig. 4b. By fitting the experimental date, constants A, B and the energy gap Δ*E* were obtained and the formula was given in Fig. 4b. The sensitivity of optical thermometry is a key parameter to value the possibility of practical applications, which is the changing rate of R in response to the variation of temperature^39^ and can be defined as:


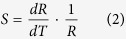


The value of sensing sensitivity as a function of temperature is displayed in Fig. 4c. The Tm^3+^-UCNCs-MT sensitivity maximum value is about 0.017 K^−1^ at 383 K.

**Figure 5:**
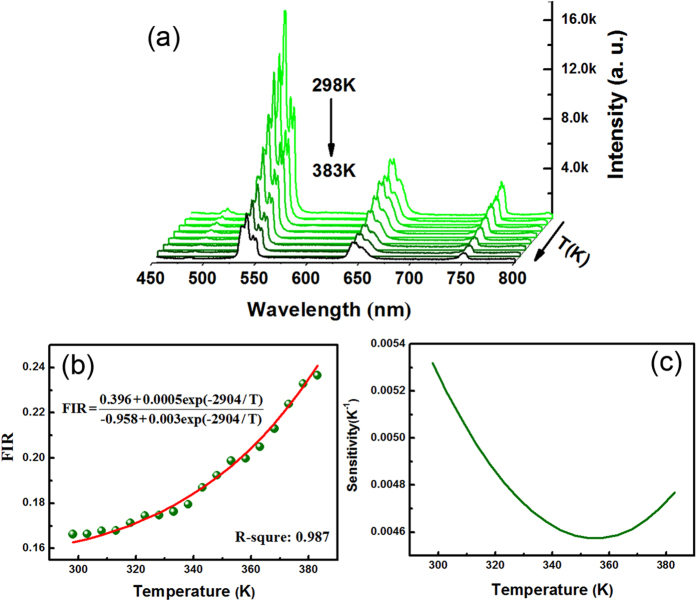
Optical-thermal properties of the Ho^3+^-UCNCs-MT. (**a**) Emission spectra of Ho^3+^-UCNCs-MT in the range from 450 to 800 nm measured at a series of temperatures between 298 and 383 K. (**b**) The relationship between the FIR and the temperature for Ho^3+^-UCNCs-MT. (**c**) The sensing sensitivity as a function of temperature for Ho^3+^-UCNCs-MT.

For a single Ho^3+^-UCNCs-MT, the spectra in the range from 450 to 800 nm are shown in Fig. 5a. The 541 nm peaks intensity (*I*_541_) reduces with rise of the temperature at a higher decay rate than that of the 750 nm peak (*I*_750_). The *I*_541_ and *I*_750_ corresponding to the energy level transition are (^5^F_4,5_/S_2_) → ^5^I_8_ and (^5^F_4,5_/S_2_) → ^5^I_7_. (see Supplementary Fig. S11) In the case of emission from two adjacent excited states to lower states, the FIR can be analyzed by using a four-level system^40^ which can be defined by the following equation^41^,^42^:


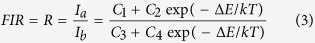


where C_1_, C_2_, C_3_ and C_4_ are constants depending on spontaneous emission rates, degeneracy and emission energies. Figure 5b shows the experimentally derived FIR of emission at 541 nm relative to emissions at 750 nm. By fitting the experimental date, one obtained the C_1_ is 0.396, C_2_ is 0.0005, C_3_ is −0.958 and C_4_ is 0.003. Figure 5c shows the optical-thermal sensing sensitivity of Ho^3+^-UCNCs-MT, the maximum value is 0.0053 K^−1^ at 298 K. It should be noted that the observed sensitivity of Tm^3+^-UCNCs-MT is 3 times higher than that of Ho^3+^-UCNCs-MT, and the sensitivity of both UCNCs-doped MT is significantly enough for thermometry applications in the range of 298–383 K.

## Discussion

In this work, we have demonstrated a simple method to dope the UCNCs in the MT by flame-heated drawing from the silica capillary tubing. The fabricated MT has an external diameter down to 2 μm, with the wall thickness of about 400 nm. The single MT was proved to be an active waveguide by guiding performance measurement. One application of the UCNCs doped MT is optical temperature sensing. As demonstrated by utilizing FIR technique, both the Tm^3+^-UCNCs-MT and the Ho^3+^-UCNCs-MT have a high sensitivity in the biophysical temperature range of 298–383 K. Moreover, the UCNCs covered by the silica MT will make it possible to excite this micro-system in flowing gas or fluid. Our work represents a first step in developing a new generation of micro-light emitting device in future integrated photonic platforms.

## Methods

### Preparation of *β*-NaLuF_4_: Yb^3+^/Tm^3+^ and *β*-NaLuF_4_:Yb^3+^/Ho^3+^ nanocrystals

In a typical synthesis of UCNCs nanocrystals, the LnCl_3_ (Ln = Lu, Yb, Tm/Ho) with rare earth ions molar ratio of 79:20:1 were added to a 100 mL three-necked flask containing 6 mL oleic acid (OA) and 15 mL 1-octadecene (ODE), The mixture was heated to 150 °C to form a pellucid solution and remove residual water and oxygen, and then cooled down to room temperature. 10 mL of methanol solution containing NaOH (2.5 mmol) and NH_4_F (4 mmol) was slowly dropped into the flask and stirred for 30 min to ensure that all fluoride was completely consumed. The solution was heated to 300 °C at a rate of about 40 °C/min, and then maintained at 300 °C for 1 h under argon atmosphere. After cooled down to room temperature, the precipitates were separated by centrifugation at 5000 rpm, washed with ethanol, and then dried in the air at 60 °C for 12 h.

### Preparation of UCNCs-MT

The UCNCs-MT was prepared by a preheat and flame-heated drawing method from silica capillary tubing (outside diameter ~162 μm, inner diameter ~100 μm, refractive index ~1.55, Polymicro Technologies, L. L. C.). The UCNCs powder was squeeze in silica capillary tubing by a fiber with diameter about 80 μm under microscope. After repeated several times, a certain amount of the UCNCs powder was placed in the middle of the tubing. Two miniature alcohol lamps were used to heat both ends of the tubing in 3 seconds, the UCNCs powder section of tubing was heated by hydrogen flame, at the same time; the tubing was drawn by the stepping motor. The MT with different diameter can be obtained by increasing or decreasing the drawing speed.

### Optical Characterization of the UCNCs-MT

The MT was deposited on a low-index MgF_2_ substrate and pumped by a 980 nm continuous laser source which was coupled in a tapered fiber. Emission of the UCNCs-doped MT was collected by a 40× objective (NA = 0.65). The pump power outgoing from the tapered fiber was measured and averaged using an optical power measurement (Thorlabs PM120D). Reflection of the pump light was removed by a 980 nm filter, and the emission of the UCNCs-doped MT was split by a beam splitter to a spectrometer (Ocean optics QE Pro) and CCD (Olympus DP 26).

## Additional Information

**How to cite this article**: Li, H. *et al*. Dispersing upconversion nanocrystals in a single silicon microtube. *Sci. Rep.* **6**, 35941; doi: 10.1038/srep35941 (2016).

## Supplementary Material

Supplementary Information
