# Supplementary material for: Dispersing upconversion nanocrystals in a single silicon microtube
Source: Sci Rep. 2016 Oct 25;6:35941. doi: 10.1038/srep35941 (PMC5078789; doi:10.1038/srep35941)
Supplement: Supplementary Information [file srep35941-s1.pdf]

Supplementary Information

Dispersing upconversion nanocrystals in a single silicon microtube

Hanyang Li[1,+], Yan Wang[1,+], Hui Li[2], Yundong Zhang[2], Jun Yang[1,*]

[1]Key Lab of In-fiber Integrated Optics, Ministry Education of China, Harbin Engineering University, Harbin 150080, China

[2]National Key Laboratory of Tunable Laser Technology, Institute of Opto-Electronics, Harbin Institute of Technology, Harbin 150080, China

[*]yangjun@hrbeu.edu.cn

[+]these authors contributed equally to this work

1. The process of the UCNCs-doped MT drawing

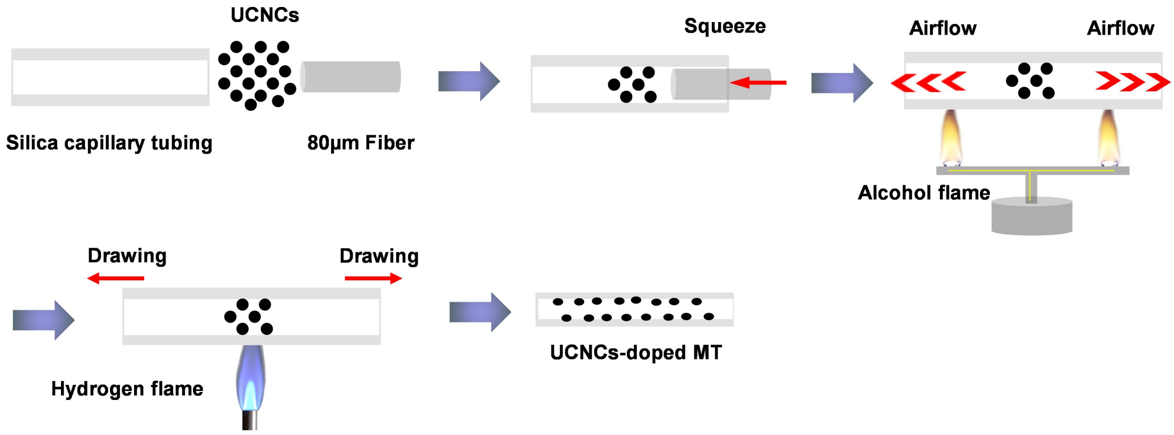

Figure S1. Schematic of UCNC-doped MT fabrication by preheat and flame-heated drawing method from silica capillary tubing.

The silica capillary tubing (outside diameter ~162 μm; inner diameter ~ 100 μm, refractive index ~ 1.55) was purchased from Polymicro Technologies, L. L. C. The polymer coating of silica capillary tubing was removed by alcohol flame heated and trichloromethane washed. The optical fiber with diameter of 80 μm (RC1310 80-21/165) was purchased from Yangtze Fiber and Cable Joint Stock, L.L.C.

The UCNCs powder was squeeze in silica capillary tubing by the 80 μm fiber under microscope. Both ends of the tubing was heat by alcohol flame in 3 seconds, then the hydrogen flame was used to heat the tubing, at the same time, the tubing was drawn by stepping motors. The MT with different diameter can be obtained by increasing or decreasing the drawing speed. In the hydrogen flame heating process, the silica capillary tubing in the molten-like state, result in the UCNCs were stick to the inner wall of the tube.

2. Schematic of experiment setup for UCNCs-MT optical properties measurement

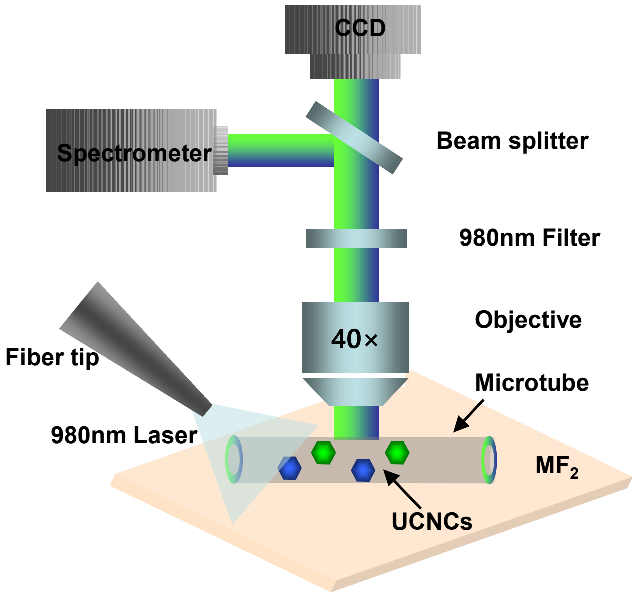

Figure S2. Schematic of the instrumental setup for the UCNCs-MT excitation experiment.

3. Characterization of UCNCs distributed in the MT

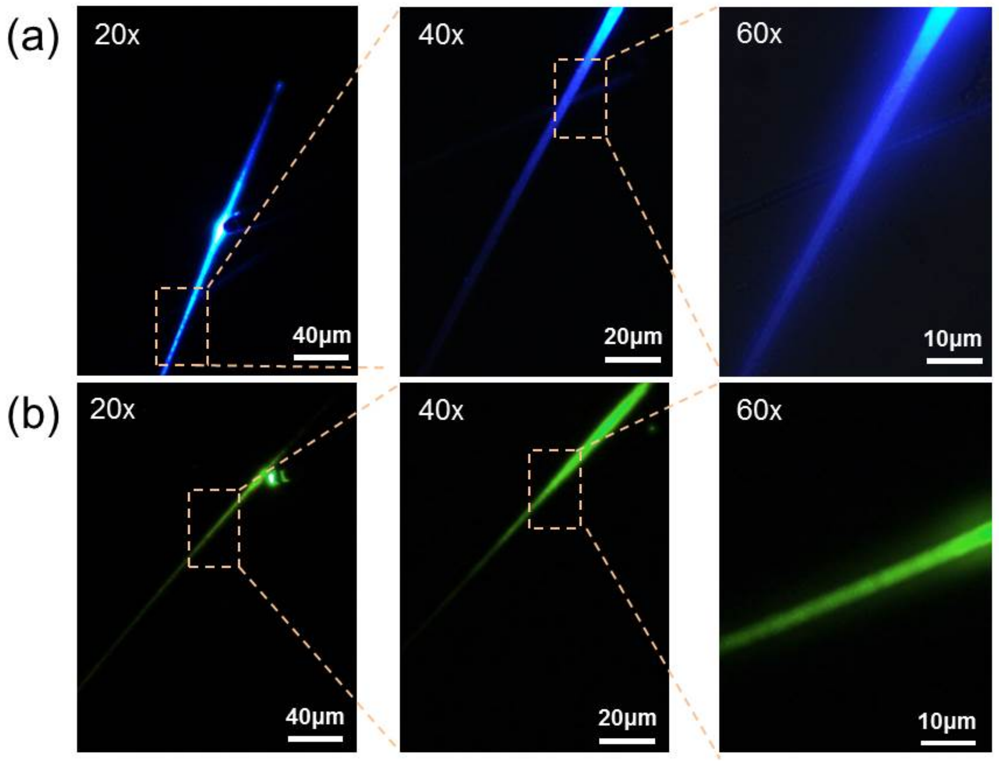

Figure S3. True-color PL microscope images of the MTs taken under 20× objective, 40× objective, and 40× objective. (a) Tm^{3+}-UCNCs-MT with diameter of 4μm. (b) Ho^{3+}-UCNCs-MT with diameter of 3μm.

One single Tm^{3+}-UCNCs-MT with diameter of 4μm and the other single Ho^{3+}-UCNCs-MT with diameter of 3μm were excited by 980nm laser. Fig. S3 shows the true-color PL microscope images of the MTs. The PL microscope image was obtained at 20× objective, then part of the MT was magnified at 40× objective, and further magnified at 60× objective. One can see that both of the MT's luminescence is uniform under the 60× objective. There is no evidence of appreciable scattering centers such as break points or UCNCs clustering.

4. Guiding performance of MT

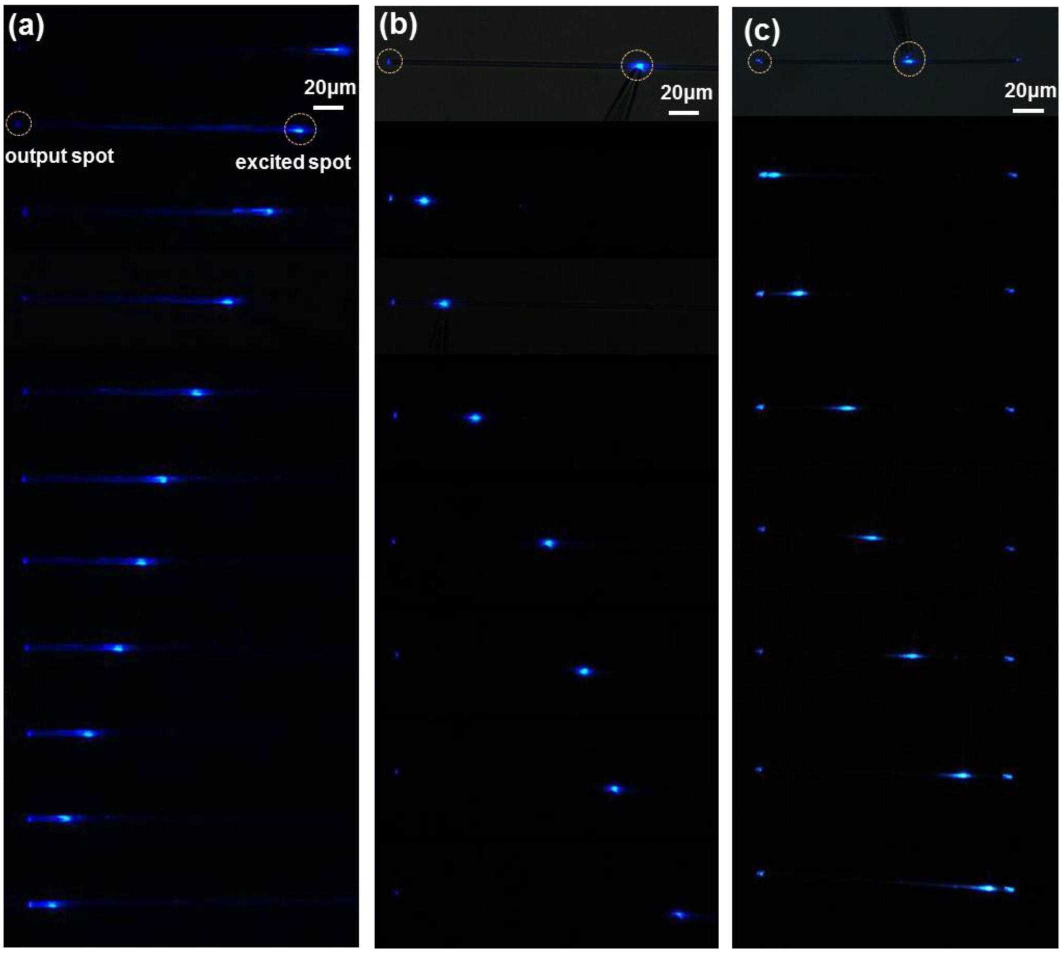

Figure S4. True-color PL microscope images of Tm^{3+}-UCNCs-MT was pumped by 980nm laser with same excitation power. (a) MT with diameter of 6μm. (b) MT with diameter of 4μm. (c) MT with diameter of 2μm.

Fig. S4a shows one single Tm^{3+}-UCNCs-MT (diameter ~ 6μm) was pumped by 980nm laser 11 times with same excitation power. The excited spot translated along the same MT. The intensities and profiles of such body brightness did not change substantially with position along the MT. By comparison, with increasing propagation length, tip-emission brightness was modified. Similar cases occurred in other single Tm^{3+}-UCNCs-MT samples, the dark field microscope images of other Tm^{3+}-UCNCs-MTs were shown in Fig. S4b (diameter ~ 4μm) and Fig. S4c (diameter ~ 2μm). By studying the image brightness, the normalized PL intensity of the emission light can thus be calculated. The PL intensity of the output spot is normalized against the excited spot, and then the decay of the guided normalized PL intensity dependent propagation distance (d) is obtained.

According to Lambert-Beer law, the normalized intensity is fit by first order exponential decay

as the red line shown in Fig. S4a; with d increases, the PL intensity decreases as $\approx \exp(-\alpha d)$, and the loss coefficient is $\alpha_1 = 101\ cm^{-1}$. The $\alpha_2 = 78\ cm^{-1}$ for Tm^{3+}-UCNCs-MT with diameter of 4μm, and $\alpha_3 = 189\ cm^{-1}$ for Tm^{3+}-UCNCs-MT with diameter of 2μm as shown in Fig. s5b and Fig. s5c, which are correspond to the MT in Fig. s4b and Fig. s4c.

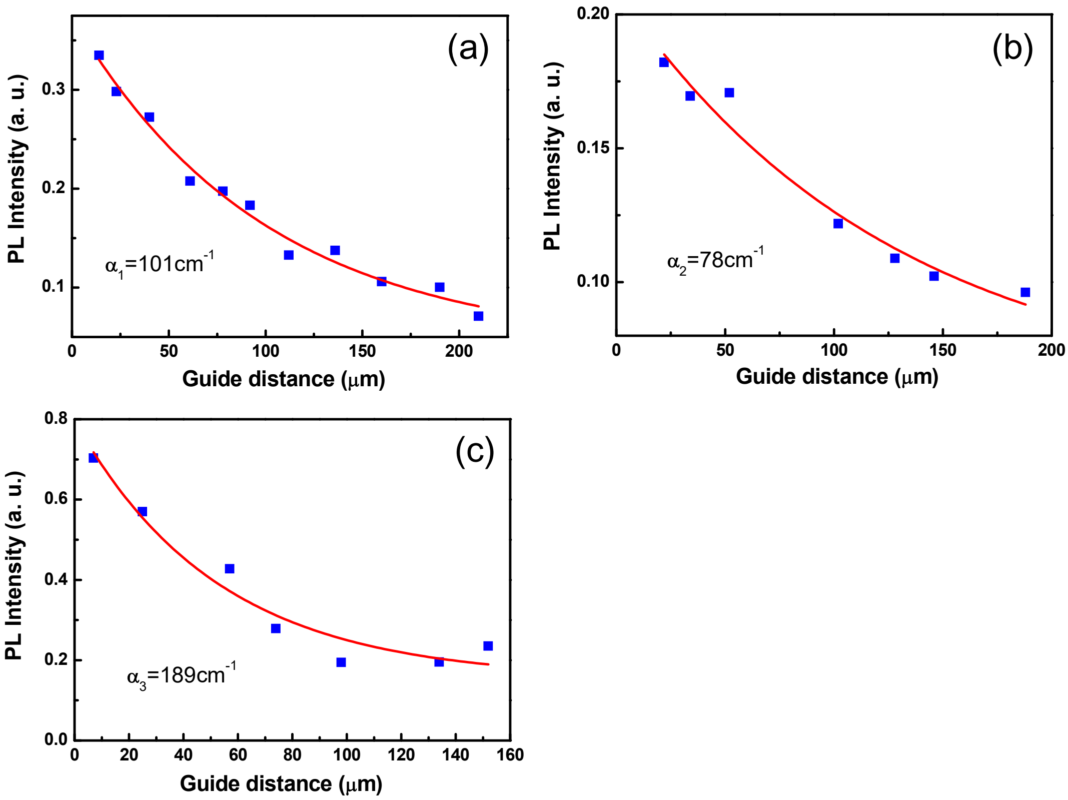

Figure S5. Normalized dependence of the MT's tip PL emission intensity on excitation location. The line is an exponential fit to the data yielding the loss coefficient α for the waveguide. (a) MT with diameter of 6μm. (b) MT with diameter of 4μm. (c) MT with diameter of 2μm

Three single Ho^{3+}-UCNCs-MT were measured by the same method. Fig. S6 shows the Dark field microscope images of Ho^{3+}-UCNCs-MT is pumped, the diameters of the MT are 4μm, 3μm, and 2μm. Corresponding loss coefficient are $\gamma_1 = 219\ cm^{-1}$, $\gamma_2 = 185\ cm^{-1}$ and $\gamma_3 = 166\ cm^{-1}$, as shown in Fig. S7.

Obviously, MT with the same UCNCs but different diameters have different loss coefficient since different diameter MTs of the scattering light is different. However, the Tm^{3+}-UCNCs-MT with diameter of 2μm has a larger $\alpha_3 = 189\ cm^{-1}$ which may be caused by the uneven surface of the MT as shown the first image of Fig. S4c. On the other hand, the loss coefficient α_3 and γ_3 have a little difference also the MTs have a same diameters (~ 2μm), which may be caused by the difference up-conversion luminescence intensity between Tm^{3+} and Ho^{3+}.

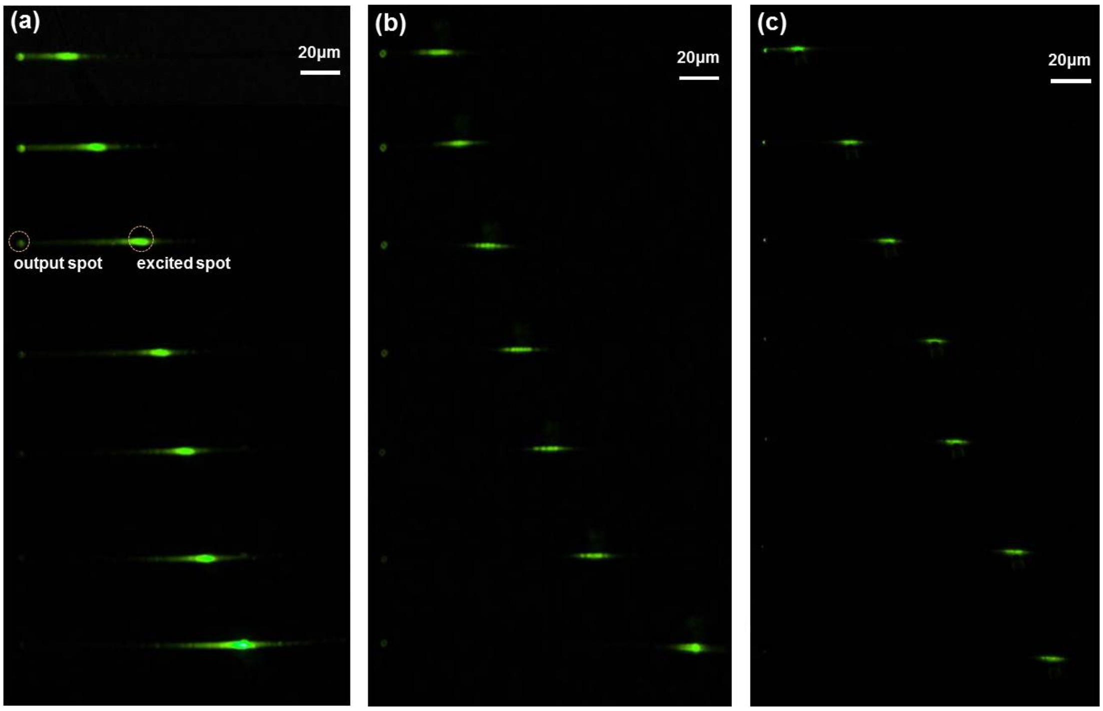

Figure S6. True-color PL microscope images of Ho^{3+}-UCNCs-MT was pumped by 980nm laser with same excitation power. (a) MT with diameter of 4μm. (b) MT with diameter of 3μm. (c) MT with diameter of 2μm.

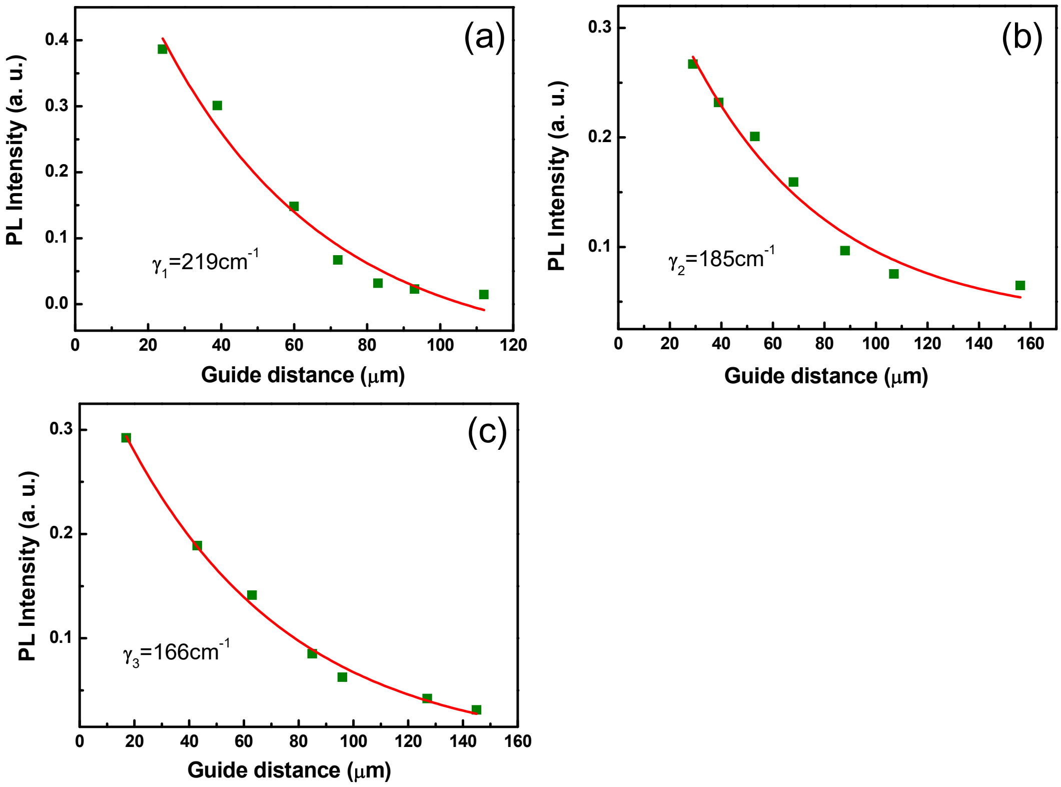

Figure S7. Normalized dependence of the MT's tip PL emission intensity on excitation location. The line is an exponential fit to the data yielding the loss coefficient γ for the waveguide. (a) MT with diameter of 4μm. (b) MT with diameter of 3μm. (c) MT with diameter of 2μm

5. Tune the cross angle between two Ho^{3+}-UCNCs-MTs

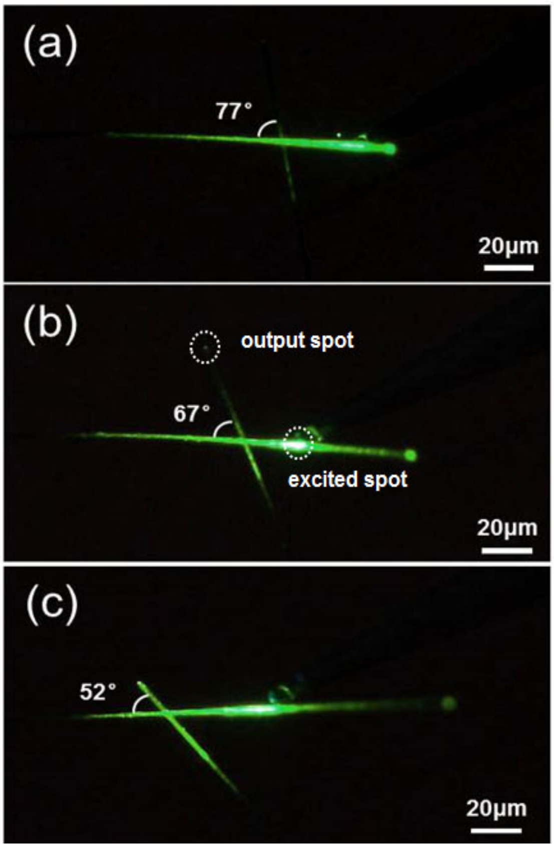

Figure S8. True-color PL microscope images of two Ho^{3+}-UCNCs-MTs. (a) Cross angle is 77°. (b) Cross angle is 67°. (c) Cross angle is 52°.

One Ho^{3+}-UCNCs-MT with diameter of 4μm was excited by the 980nm laser fiber tip, another MT with diameter of 2μm which is placed underneath. Figure S8 shows when the cross angle is 77° between two MT and the coupling efficiency is very low. We estimate the coupling efficiency by studying the image brightness and normalized PL intensity of the emission light. Here we defined as coupling efficiency $\eta = I_{\text{output spot}} / I_{\text{excited spot}}$, and the estimated η is about 3.9%. When the cross angle is tune to 67° the estimated η is about 6.1%. When the cross angle is tune 52° the estimated η is about 21.4%. We found that tune the cross angle between two MT could make the coupling more prominent.

6. The energy distribution and photon transition of Tm^{3+}-UCNCs-MT

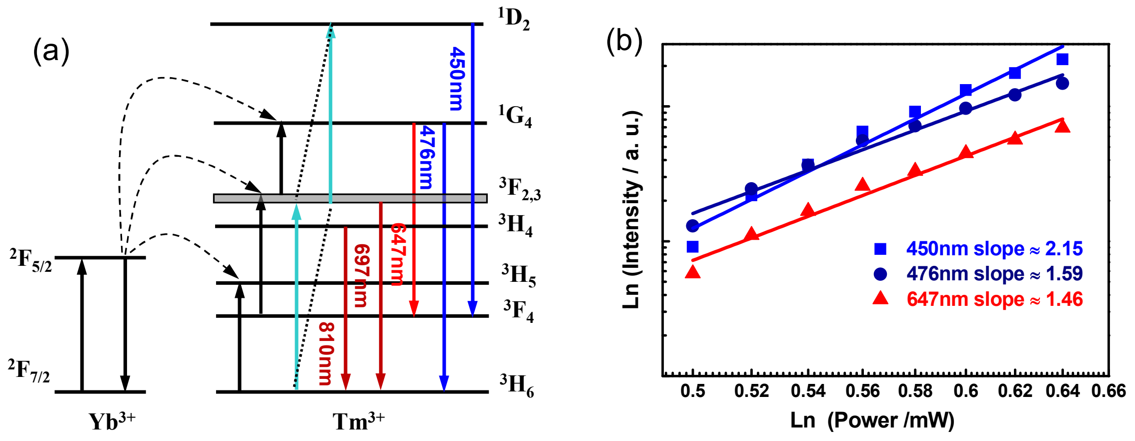

Figure S9. (a) Energy level diagram of Tm^{3+}-UCNCs-MT and possible up-conversion processes. (b) Ln – Ln excitation power dependence of up-conversion emissions intensities for Tm^{3+}-UCNCs-MT.

The PL spectra of Tm^{3+}-UCNCs-MT (shown in Fig. 3c) consist of five emission peaks at 450 nm, 476 nm, 647nm, 697 nm, and 810 nm, which are assigned to the $^1D_2 \rightarrow {}^3F_4$ (450 nm), $^1G_4 \rightarrow {}^3H_6$ (476 nm), $^1G_4 \rightarrow {}^3F_4$ (647 nm), $^3F_{2,3} \rightarrow {}^3H_6$ (697 nm), and $^3H_4 \rightarrow {}^3H_6$ (810 nm) transitions, respectively. The number of photons which are required to populate the upper emitting level can be obtained by the relation $I_f \propto P^n$. We measured the emissions intensities of Tm^{3+}-UCNCs-MT under different excitation power, the experimental data were fitted with a straight line and slope value was obtained as shown in Fig. S9b.

7. One single Tm^{3+}-UCNCs-MT was pumped in different excitation power

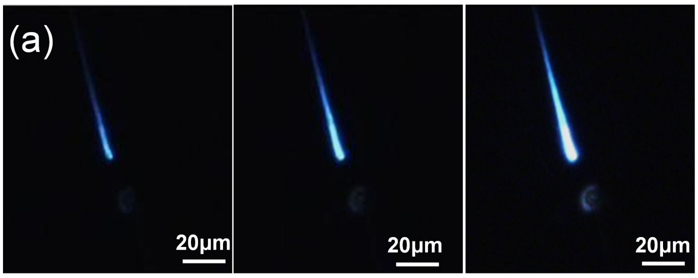

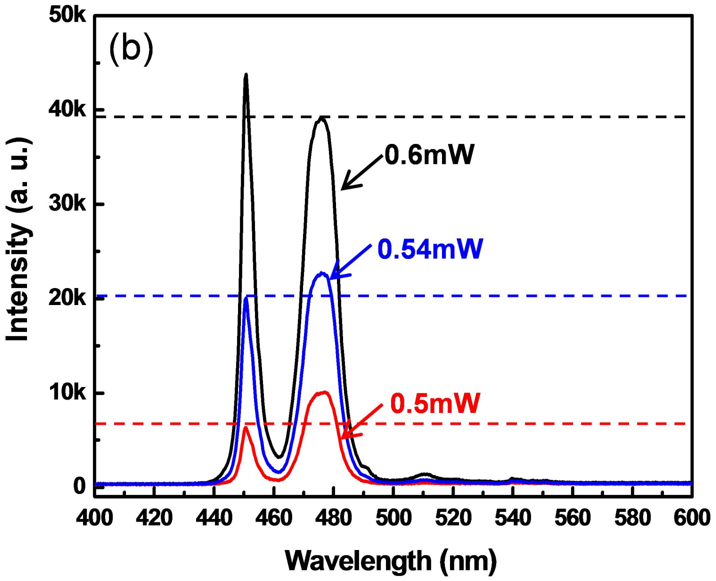

Figure S10. (a) True-color PL microscope images of single Tm^{3+}-UCNCs-MT with diameter of 5μm and length of 100μm which was pumped in different excitation power. (b) The corresponding emission spectra.

One single Tm^{3+}-UCNCs-MT (diameter ~5 μm, lengths ~ 100 μm) was pumped with the non-contact mode excitation way. The excitation laser power was 0.5mW, 0.54mW, and 0.6 mW. At relatively low excitation power, for example 0.5 mW and 0.54 mW, the up-conversion luminescence is 2 absorbed photons process, the peak of λ = 450nm is smaller than peak of λ = 476 nm. At relatively high excitation power, for example 0.6mW, the up-conversion luminescence is 3 absorbed photons process, the peak of λ = 450nm is greater than peak of λ = 476nm as shown in Fig. S10. However, for smaller diameter Tm^{3+}-UCNCs-MT (diameter ~2 μm), pump with the contact mode excitation way is same to increase pump power density, the up-conversion luminescence at λ= 450 nm could be 3 photons process as shown in Fig. 3d.

8. The energy distribution and photon transition of Ho^{3+}-UCNCs-MT

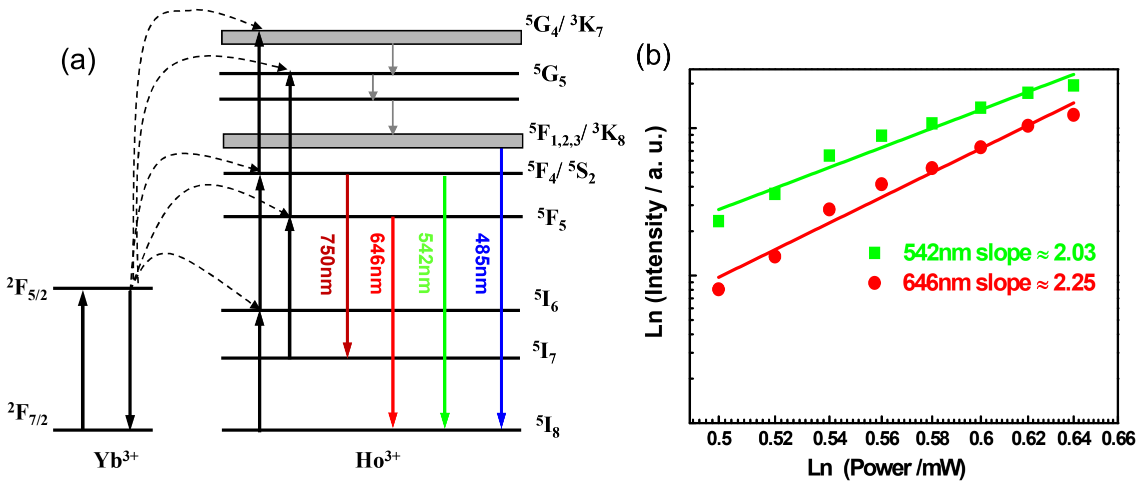

Figure S11. (a) Energy level diagram of Ho^{3+}-UCNCs-MT and possible up-conversion processes. (b) Ln–Ln excitation power dependence of up-conversion emissions intensities for Ho^{3+}-UCNCs-MT.

The PL spectra of Ho^{3+}-UCNCs-MT (shown in Figure 5a) consist of four emission peaks at 485 nm, 542 nm, 646nm, and 750nm, which are assigned to the $^5F_{1,2,3}/^3K_8 \rightarrow {}^5I_8$ (485 nm), $^5F_4/^5S_2 \rightarrow {}^5I_8$ (542 nm), $^5F_5 \rightarrow {}^5I_8$ (646 nm) and $^5F_4/^5S_2 \rightarrow {}^5I_7$ (750 nm) transitions, respectively. The slope of the emissions and Ln -Ln relationship between excitation power and emissions intensities were shown in Fig. S11.
